# Ganghwaljetongyeum, an anti-arthritic remedy, attenuates synoviocyte proliferation and reduces the production of proinflammatory mediators in macrophages: the therapeutic effect of GHJTY on rheumatoid arthritis

**DOI:** 10.1186/1472-6882-13-47

**Published:** 2013-02-26

**Authors:** Bo-Ram Jeoung, Kyung Dong Lee, Chang-Su Na, Young-Eok Kim, BoA Kim, Young Ran Kim

**Affiliations:** 1 Department of Pharmaceutical Engineering, Dongshin University, Naju, Jeonnam, 520-724, South Korea; 2 Department of Oriental Medicine Materials, Dongshin University, Naju, Jeonnam, 520-724, South Korea; 3 College of Oriental Medicine, Dongshin University, Naju, Jeonnam, 520-724, South Korea; 4 Department of Physical Therapy, Dongshin University, Naju, Jeonnam, 520-724, South Korea; 5 Department of Microbiology, Chonnam University Medical School, Gwangju, 501-746, South Korea

**Keywords:** Ganghwaljetongyeum, Rheumatoid arthritis, Synoviocytes, Inflammation, IL-1β, NF-κB

## Abstract

**Background:**

Ganghwaljetongyeum (GHJTY), a complex herbal decoction, is used to treat rheumatoid arthritis. However, the action mechanism of GHJTY is not still unclear on rheumatoid arthritis. In this study, we examined the beneficial effects and the action mechanisms of GHJTY on synoviocyte proliferation and inflammatory mediators.

**Methods:**

To test the effect of GHJTY on synoviocyte proliferation, HIG-82 cells, rabbit knee synovial membrane cells, were treated with GHJTY under IL-1β. To evaluate the effects of GHJTY on proinflammatory mediators, we tested cytokine levels in RAW264.7 cells.

**Results:**

Proliferation of HIG-82 cells was significantly inhibited by GHJTY treatment. We found that GHJTY caused cytoskeleton damage to HIG-82 cells. In contrast, treatment of GHJTY did not show any cytotoxicity to other different origin cell lines, HeLa and RAW264.7 cells. GHJTY inhibited IL-1β-mediated NF-κB activation in HIG-82 cells and reduced the LPS-mediated production of proinflammatory cytokines, TNF-α, IL-12, and NO in RAW264.7 cells. In addition, the expression of cyclooxygenase in LPS-activated RAW264.7 cells was also decreased by GHJTY treatment.

**Conclusions:**

These results suggest that GHJTY might effectively attenuate rheumatoid arthritis by inhibiting the production of proinflammatory mediators and the proliferation of synoviocytes.

## Background

Rheumatoid arthritis is a chronic inflammatory disorder accompanied by hyperplasia of the cartilage lining caused by infiltration of inflammatory cells, ultimately resulting in joint damage [1,2]. One of the most striking features of inflammatory arthritis is hyperplasia of synovial fibroblasts. Synovitis was reported to play an essential role in the pathophysiology of rheumatoid arthritis [3,4]. The progression of rheumatoid arthritis is accelerated by proinflammatory cytokines and chemokines, and tumor necrosis factor-α (TNF-α) and interleukin-1β (IL-1β) are reported to be associated with the progression of rheumatoid arthritis [5]. In response to these cytokines, synovial fibroblasts proliferate vigorously and form pannus tissues, which destroy the cartilage and bone of the joints [6-8]. Nuclear factor-κB (NF-κB) is a transcription factor which regulates gene expression associated with inflammation and is strongly activated by a range of stimuli including TNF-α, IL-1β, LPS, UV light, and oxidative stress [9]. Cyclooxygenase (COX) is strongly induced by IL-1β and plays an important role in the pathophysiology of rheumatoid arthritis [10]. Therefore, down-regulation of proinflammatory cytokines and NF-κB may be an appropriate therapeutic strategy for rheumatoid arthritis. Antiproliferative agents that modulate synoviocyte growth have been suggested to have potential as anti-rheumatic drugs.

Pain-relieving and nonsteroidal anti-inflammatory drugs (NSAIDs) can help to reduce pain and inflammation in rheumatoid arthritis patients. New approach to the treatment of rheumatoid arthritis has been concentrated to develop safe and potent drugs. Some oriental medicines [11-13] have been used to treat rheumatoid arthritis. Curcumin, a natural product was reported to inhibit inflammatory processes associated with arthritis [14]. Gangwhaljetongyeum (GHJTY), a traditional Korean herbal medicine comprised with several component herbs (Table 1), has been used to treat severe joint pain, limitation of motion, fever, and swelling. In this study, we attempted to evaluate the remedial value and the action mechanism of GHJTY on rheumatoid arthritis by testing its effect on the production of proinflammatory mediators and the proliferation of synoviocytes.

**Table 1:** Prescription of GHJTY in daily doses

**Drug names**	**Botanical origin**	**Weight (g)**	**Source**
*Angelicae Koreanae Radix*	*Angelica Koreanum* (Umbelliferae)	6	Korea
*Atractylodis Rhizoma*	*Atractylodes chinensis* (Compositae)	6	China
*Araliae Continentalis Radix*	*Aralia continentalis *(Araliaceae)	4	Korea
*Paeonia Radix Rubra*	*Paeonia obovata* (Paeoniaceae)	4	Korea
*Stephaniae Tetrandrae Radix*	*Sinomenium acutum* (Menispermaceae)	4	China
*Clematidis Radix*	*Clematis mandshurica* (Ranunculaceae)	4	China
*Angelicae Gigantis Radix*	*Angelica gigas* (Umbelliferae)	4	Korea
*Hoelen*	*Poria cocos *(Polyporaceae)	4	China
*Alismatis Rhizoma*	*Alisma orientale *(Alismataceae)	4	Korea
*Akebiae Caulis*	*Akebia quinata* (Lardizabalaceae)	4	Korea
*Citri Pericarpium*	*Citrus unshiu *(Rutaceae)	4	Korea
*Chaenomelis Fructus*	*Chaenomeles sinensis *(Rosaceae)	4	Korea
*Phellodendri Cortex*	*Phellodendron amurense *(Rutaceae)	3	China
*Glycyrrhizae Radix*	*Glycyrrhiza uralensis *(Leguminosae)	2	China
*Juncus Medulla*	*Juncus effuses *(Juncaceae)	4	China
*Gleditsiae Spina*	*Gleditsia sinensis *(Leguminosae)	4	China
*Lonicerae Caulis*	*Lonicera japonica *(Caprifoliaceae)	4	China
*Taraxaci Herba*	*Taraxacum platycarpum *(Compositae)	4	Korea
**Total amount**		**73**	

## Methods

### Preparation of GHJTY extract

Herbal medicines used in this study were purchased from Hwalim Natural Drug Co. Ltd (Busan, Korea) and the origins were described in Table 1. The herbal formula of GHJTY (Table 1) was extracted with hot distilled water at 100°C for 2 hours at Dongshin University Oriental Hospital (Gwangju, Korea). The extract was centrifuged at 1,500 rpm for 20 min and the supernatant was concentrated by evaporation under vacuum with preferably low temperature (EYELA, Japan). The concentrated extract was lyophilized by a vacuum freeze drier at −80°C (Samwon Freezing Eengineering Co., Korea).

### Cell cultures

A rabbit knee synovial membrane cell line, HIG-82 (American Type Culture Collection, Manassas, VA, USA), was cultured in Ham’s F-12 nutrient mix medium (Ham’s F-12, GIBCO, Invitrogen, Carlsbad, CA, USA) supplemented with penicillin-streptomycin and 10% fatal bovine serum (FBS, GIBCO, Invitrogen) under an atmosphere of 5% CO_2_ in a humidified incubator. HeLa and RAW264.7 cells (Korea Cell LineBank, Korea) were maintained in DMEM (GIBCO, Invitrogen) and RPMI (GIBCO, Invitrogen), respectively under above cell culture conditions.

### Effect of GHJTY on cell proliferation

Cells seeded in a 96-well microplate (Corning, NY, USA) at a density of 2 × 10^4^ cells/well were cultured with or without IL-1β (5 ng/ml) (Sigma-Aldrich, Saint Louis, Missouri, USA). Celecoxib (Sigma-Aldrich) was used as a control drug of arthritis. GHJTY or celecoxib was added to the cells for 2 days. Cell proliferation was assayed using 3-(4,5-dimethylthiazol-2-yl)-5-(3-carboxymethoxyphenyl)-2-(4-sulfophenyl)-2H-tetrazolium (MTS), according to manufacturer’s instructions (Promega, Madison, WI, USA). Absorbance was read by an ELISA microplate reader (Power Wavex340, NIO-TEK-INS TRUMENTS, INC) at 490 nm. Proliferation was calculated as the percentage (%) to not-treated cells.

### Effect of GHJTY on actin cytoskeleton of HIG-82 cells

HIG-82 cells were seeded in an 8-well glass chamber plate (Nalge Nunc International) at a density of 3 × 10^4^ cells/well and cultured with or without IL-1β (5 ng/ml) in Ham’s F-12 with 10% FBS for 2 days. After washing with HBSS (GIBCO, Invitrogen), the cells were fixed in 3.7% formaldehyde for 15 min, permeabilized with 0.1% Triton X-100 (Sigma), and incubated in a blocking solution of Image iT signal enhancer (Molecular Probes, Invitrogen) for 30 min. F actin was visualized by Alexa Fluor 594-conjugated Phalloidin (Molecular Probes) as described previously [15]. Confocal images of specimens mounted with a ProLong gold antifade reagent with DAPI (Molecular probes) were acquired using a laser scanning confocal microscope (EZ-C1, Nikon).

### Effect of GHJTY on IL-1β-induced NF-κB transcription activity in HIG-82 cells

HIG-82 cells seeded in a 96-well plate (Corning) at 1×10^4^/well were transfected with a reporter plasmid, pNF-κB-luciferase [16] using Hily Max (Dojindo, Japan). One day after transfection, the cells were replaced with fresh Ham’s F-12 containing different concentrations of test agents and were incubated for 6 hours. The cells were treated with lysis buffer (Promega) and luciferase activities were assayed by a luminometer (MicroLumat Plus LB96V; Berthold, Wilbad, Germany).

### Effect of GHJTY on the production of nitric oxide (NO) and proinflammatory cytokines in RAW264.7 cells

RAW264.7 cells seeded at 1×10^5^/well in a 48-well plate (Corning) were preincubated with GHJTY or celecoxib for 2 hours. LPS (Sigma) was added to the cells for 20 hours. The levels of cytokines, TNF-α and IL-12 were measured in the cell supernatants using sandwich ELISA kits following the manufacturer’s experimental protocols (Biolegend, USA). Absorbance was read by an ELISA microplate reader (Power Wavex340, NIO-TEK-INS TRUMENTS, INC) at 450 nm. NO in the culture supernatant was measured using Griess Reagent (0.1% N-(1-naphthyl)-ethylendiamine dihydrochoride, 1% Sulfanilamide in 2.5% H_3_PO_4_).

### Effect of GHJTY on the expression of COX in LPS-activated RAW264.7 cells

RAW264.7 cells were cultured in a 6-well plate (Corning, USA) at 1×10^6^/well overnight. GHJTY or celecoxib was pretreated to the cells for 2 hours and LPS (1 μg/ml) was added for 18 hours. COX protein was detected by Western blot analysis using an antibody specific to COX-1 (Santa Cruz, USA) and a detection kit, Immobilon Western (Millipore, USA). The relative amount of COX-1 protein was analyzed by LAS-4000 mini (Fujifilm, Japan).

### Statistical analyses

The results are expressed as mean ± SEM unless otherwise stated. Statistical differences were evaluated using Student's *t*-test, with a *P* value < 0.05 considered significant.

## Results and discussion

### GHJTY suppresses the proliferation of HIG-82 cells

**Figure 1:**
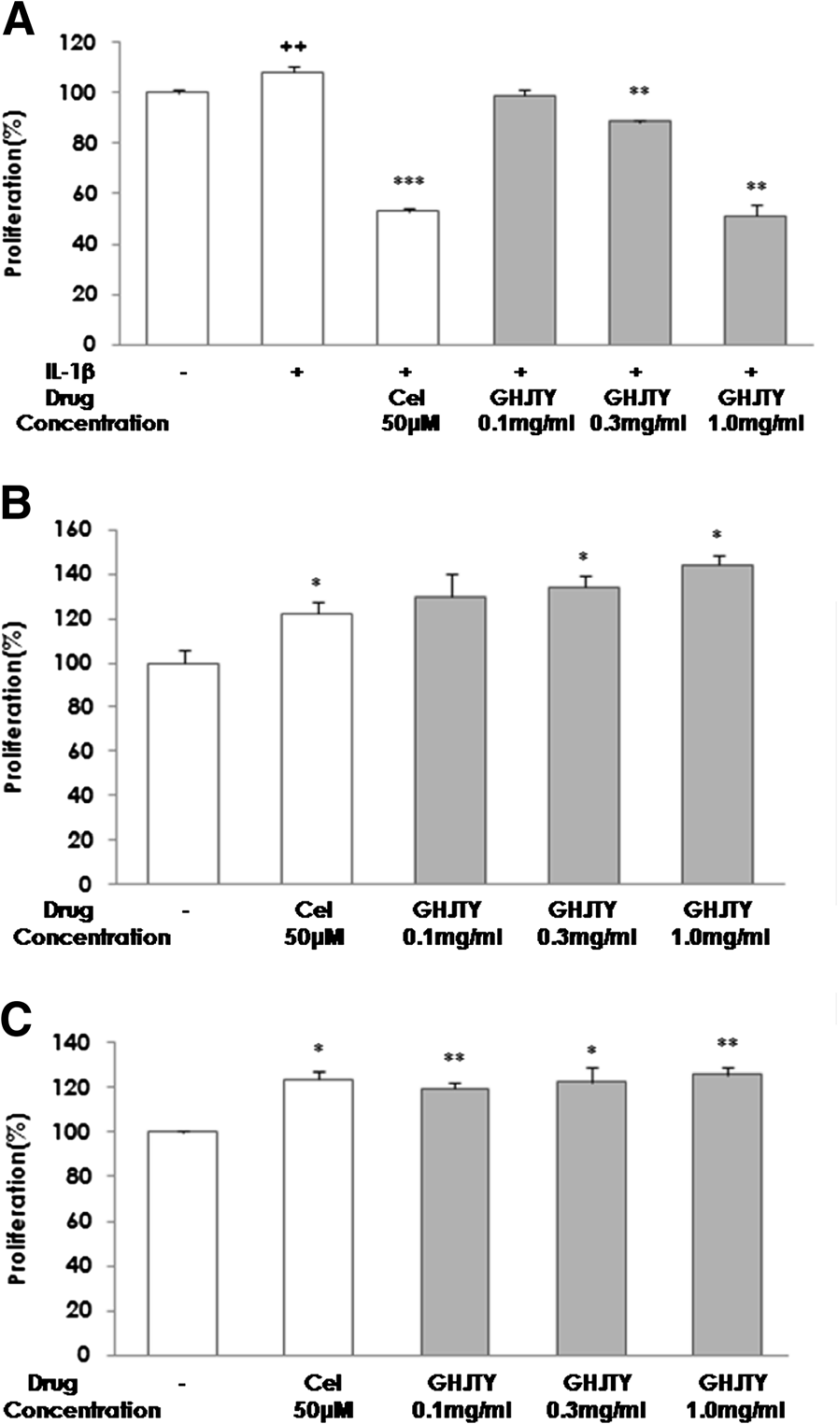
**GHJTY specifically inhibits the proliferation of HIG-82 cells.** (**A**) HIG-82 cells were exposed to IL-1β or GHJTY for 2 days and cell proliferation was assayed by MTS. Proliferation of HIG-82 cells was decreased by Celecoxib (Cel). GHJTY significantly reduced the proliferation of HIG-82 cells at concentrations of 0.3 to 1.0 mg/ml. GHJTY did not show any cytotoxic effect on other origin cells, HeLa and RAW264.7 cells (**B** and **C**). ++ *P* < 0.01: compared with non-treated group, * *P* < 0.05, ** *P* < 0.01, and *** *P* < 0.001: compared with IL-1β treated group.

To assess the effect of GHJTY on synoviocyte proliferation, HIG-82 cells were exposed to IL-1β and GHJTY for 2 days. Cell proliferation was assayed by MTS. IL-1β caused proliferation of HIG-82 cells, which was inhibited by Celecoxib, a non-steroidal anti-inflammatory drug used for the treatment of rheumatoid arthritis. GHJTY significantly suppressed the proliferation of HIG-82 cells at concentrations of 0.3 and 1.0 mg/ml (Figure 1A). In contrast, GHJTY did not show any cytotoxic effect on other origin cells, HeLa and RAW264.7 cells (Figure 1B and 1C). This result suggests that GHJTY has specific cytotoxic activity to synoviocyte HIG-82 cells.

### GHJTY causes rearrangement of actin cytoskeleton of HIG-82 cells

**Figure 2:**
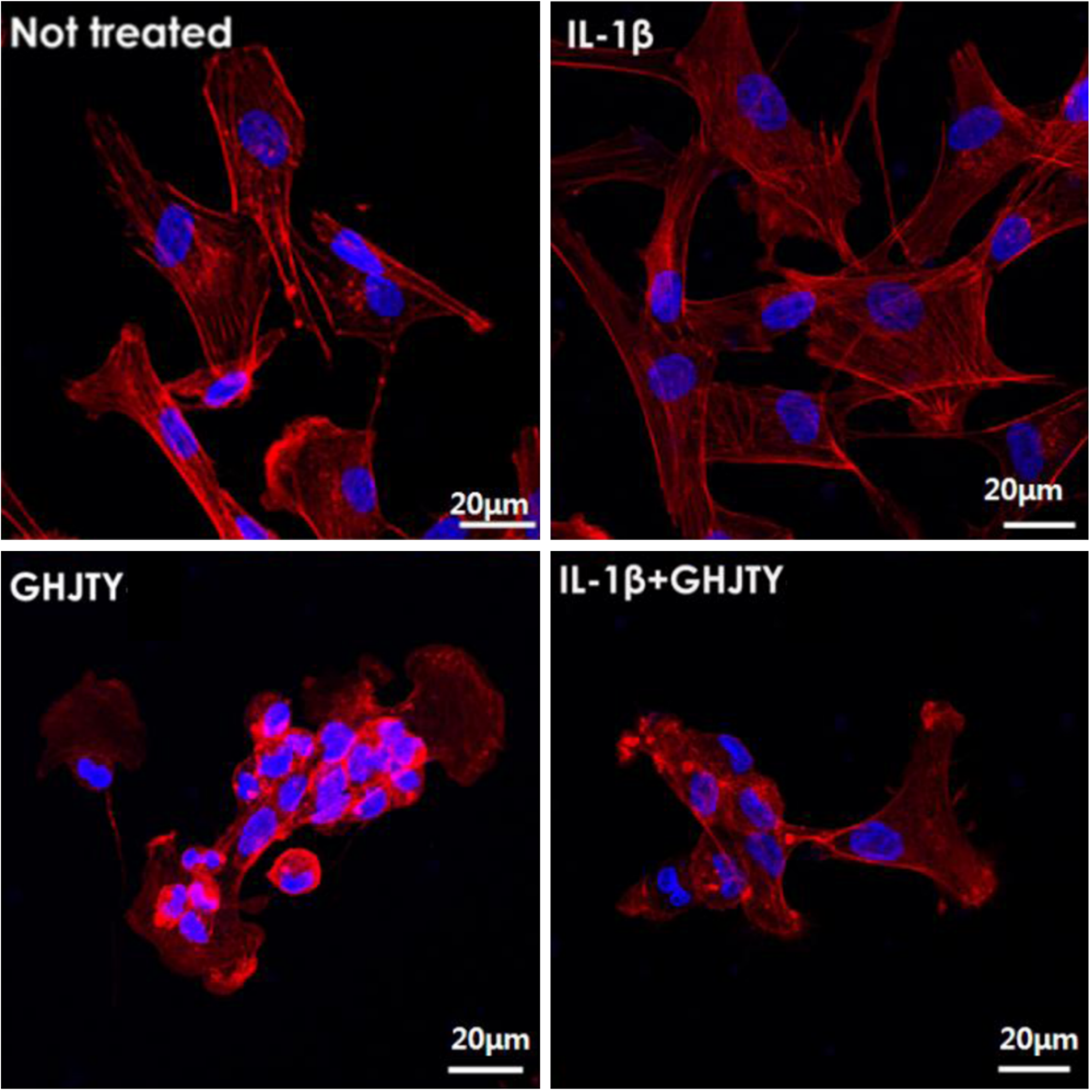
**GHJTY causes the rearrangement of actin cytoskeleton in HIG-82 cells.** HIG-82 cells were treated with GHJTY for 2 days and actin and nucleus were stained with Alexa Fluor 594-conjugated Phalloidin (red color) and DAPI (blue color), respectively. GHJTY caused the disruption of actin filament in HIG-82 cells and the nuclei of the cells to be condensed. The effect of GHJTY was also shown in IL-1β-activated HIG-82 cells.

While studying the cytotoxic effect of GHJTY on HIG-82 cells, we found that the dying cells became rounded and shrunk. To visualize the morphologic changes of HIG-82 cells, actin and nucleus were stained with Alexa Fluor 594-conjugated Phalloidin and DAPI, respectively. GHJTY caused disruption of actin filaments in HIG-82 cells (Figure 2). Nuclei of HIG-82 cells treated with GHJTY were shown to be condensed (Figure 2). The effect of GHJTY was also shown in IL-1β-activated HIG-82 cells (Figure 2).

### GHJTY suppresses IL-1β-induced NF-κB activation in HIG-82 cells

**Figure 3:**
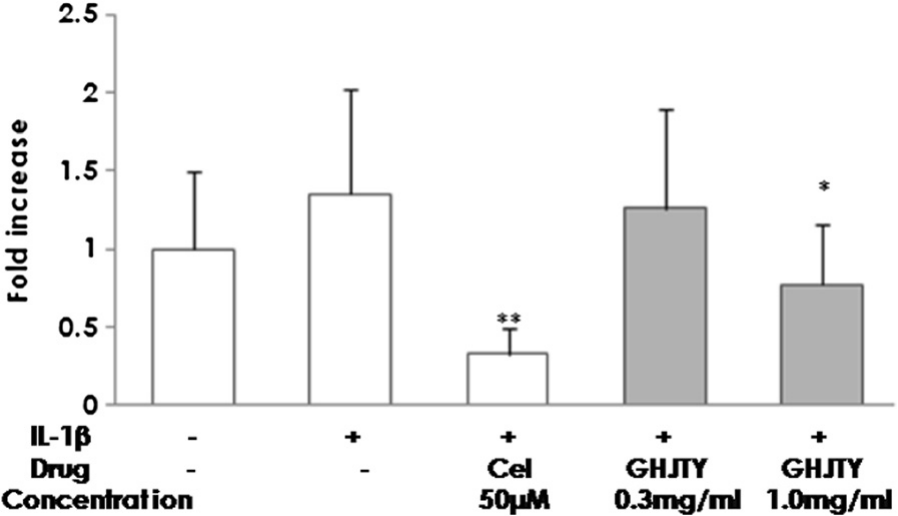
**GHJTY reduces the IL-1β-induced NF-κB activation in HIG-82 cells.** HIG-82 cells were transfected with a reporter plasmid, pNF-κB-luciferase. IL-1β increased NF- κB transcription, which was blocked by GHJTY at a concentration of 1.0 mg/ml. Celecoxib (Cel) also blocked IL-1β-induced NF-κB activation in HIG-82 cells. * *P* < 0.05 and ** *P* < 0.01: compared with IL-1β treated group.

NF-κB is a transcription factor involved in inflammation and arthritis. The effect of GHJTY was tested on NF-κB activation in HIG-82 cells. IL-1β increased NF-κB transcription, which was blocked by GHJTY at a concentration of 1.0 mg/ml (Figure 3). Celecoxib also blocked the IL-1β-induced NF-κB activation in HIG-82 cells.

### GHJTY inhibits LPS-induced production of NO, TNF-α, and IL-12 in RAW264.7 cells

**Figure 4:**
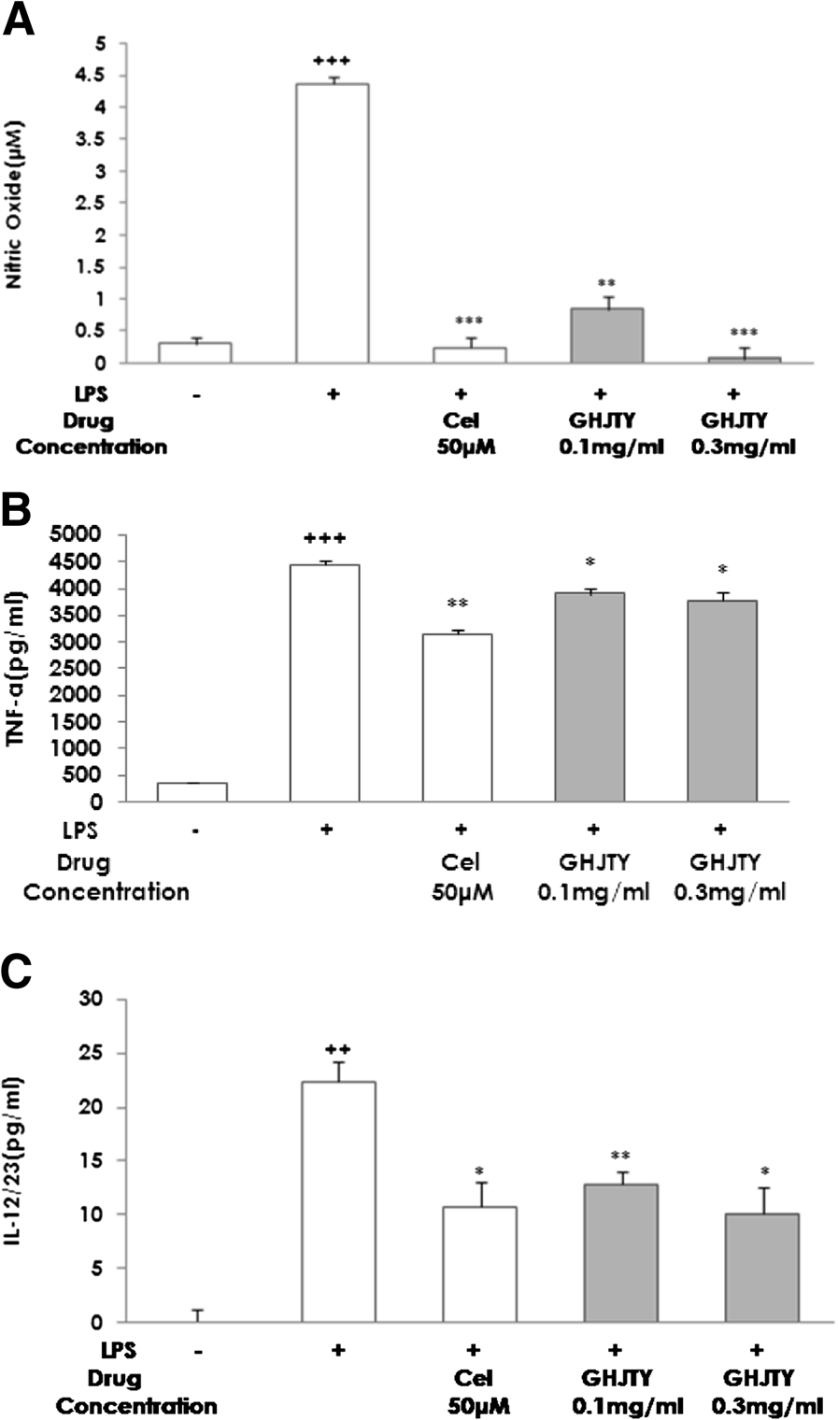
**GHJTY decreases the LPS-increased production of NO, TNF-α, and IL-12 in RAW264.7 cells.** RAW264.7 cells were pretreated with GHJTY for 2 hours and then incubated with LPS for 18 hours. (**A**) LPS-induced NO production in RAW264.7 cells was blocked by GHJTY pretreatment. Inflammatory cytokines were measured in the cell supernatants by ELISA. GHJTY decreased the LPS-induced production of TNF-α (**B**) and IL-12 (**C**) cytokines. ++ *P* < 0.01 and +++ *P* < 0.001: compared with non-treated group, * *P* < 0.05, ** *P* < 0.01, and *** *P* < 0.001: compared with LPS-treated group.

To study the effect of GHJTY on the production of inflammatory mediators, RAW264.7 cells were pretreated with GHJTY for 2 hours and then incubated with LPS for 20 hours. The LPS-induced NO production in RAW264.7 cells was significantly blocked by pretreatment with GHJTY (Figure 4A). In addition, inflammatory cytokines were measured in the cell supernatants by ELISA. GHJTY partly decreased the LPS-induced production of TNF-α and IL-12 cytokines (Figure 4B and 4C).

### GHJTY suppresses the COX-1 expression in LPS-activated RAW264.7 cells

**Figure 5:**
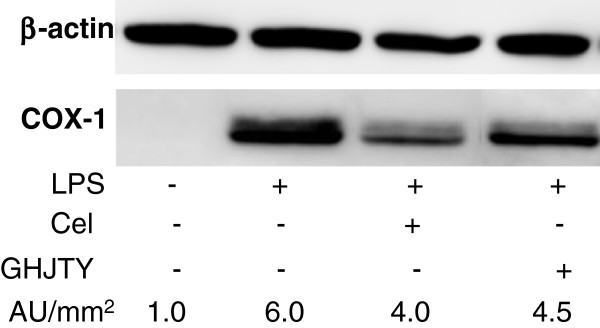
**GHJTY reduces the expression of COX-1 in LPS-activated RAW264.7 cells.** RAW264.7 cells were pretreated with GHJTY for 2 hours and LPS was added to the cells for 18 hours. β-actin was used as a control protein. COX-1 was detected by Western blot analysis and the relative amounts were described as AU/mm^2^. Celecoxib (Cel) and GHJTY reduced the expression of COX-1 in LPS-activated RAW264.7 cells.

To study the effect on COX-1 expression, GHJTY was treated to the cells for 2 hours and LPS was added for 18 hours. LPS induced COX-1 expression, which was reduced by treatment of Celecoxib, a COX inhibitor (Figure 5). GHJTY suppressed the expression of COX-1 in LPS-activated RAW264.7 cells (Figure 5).

## Conclusion

Rheumatoid arthritis is an autoimmune disease characterized by synovial proliferation, infiltration of lymphocytes and macrophage into the synovial lining, and a paucity of apoptosis [6]. We evaluated the remedial value of GHJTY, a prescription for rheumatoid arthritis, by examining its effect on synoviocyte proliferation and inflammatory responses. First, GHJTY significantly inhibited the proliferation of HIG-82 cells, a rabbit knee synovial membrane cell line (Figure 1A) by causing cytoskeleton damage to the cells (Figure 2). In contrast, GHJTY did not show any cytotoxic effects on other origin cell lines, HeLa and RAW264.7 cells (Figure 1B and 1C). In addition to the inhibitory effect on synoviocyte growth, GHJTY reduced the IL-1β-mediated NF-κB activation in HIG-82 cells (Figure 3). Moreover, GHJTY decreased the LPS-mediated production of proinflammatory cytokines, TNF-α, IL-12, and NO in RAW264.7 cells (Figure 4A, 4B, and 4C). The expression of COX-1 in LPS-activated RAW264.7 cells was also reduced by GHJTY treatment (Figure 5). Consequently, GHJTY was shown to be effective in decreasing the hyperplasia of synovial fibroblasts and inflammatory responses, the most striking features of inflammatory arthritis. These results suggest that GHJTY might effectively attenuate rheumatoid arthritis by inhibiting the production of proinflammatory mediators from macrophage-like cells and the proliferation of synoviocytes.

## Abbreviations

GHJTY: Ganghwaljetongyeum; IL-1β: Interleukine-1β; NF-κB: Nuclear factor- κB; LPS: Lipopolysaccharide; TNF-α: Tumor necrosis factor-α; IL-12: Interleukine-12; NO: Nitric oxide; COX-1: Cyclooxygenase 1; NSAIDs: Nonsteroidal anti-inflammatory drugs; Cel: Celecoxib.

## Competing interests

The authors have no competing interests.

## Authors’ contributions

BJ, KDL, and BK carried out all the assays. CN and YK participated in the design of the study and performed the statistical analysis. YRK conceived of the study, and participated in its design and coordination and helped to draft the manuscript. All authors read and approved the final manuscript.

## Pre-publication history

The pre-publication history for this paper can be accessed here:

http://www.biomedcentral.com/1472-6882/13/47/prepub
